# Morphology of Microchips in the Surface Finishing Process Utilizing Abrasive Films

**DOI:** 10.3390/ma17030688

**Published:** 2024-01-31

**Authors:** Katarzyna Tandecka, Wojciech Kacalak, Maciej Wiliński, Michał Wieczorowski, Thomas G. Mathia

**Affiliations:** 1 Department of Engineering and Informatics Systems, Faculty of Mechanical Engineering, Koszalin University of Technology, 75-620 Koszalin, Poland; wojciech.kacalak@tu.koszalin.pl (W.K.); wilinskimaciej@wp.pl (M.W.); 2 Faculty of Mechanical Engineering, Institute of Applied Mechanics, Poznan University of Technology, 3 Piotrowo St., 60-965 Poznan, Poland; michal.wieczorowski@put.poznan.pl; 3 Laboratoire de Tribologie et Dynamique des Systemes (LTDS), Ecole Centrale de Lyon, Centre National de la Recherche Scientifique, 69134 Lyon, France; thomas.mathia@ec-lyon.fr

**Keywords:** surface finishing, lapping film, finishing, generation of microchips, morphology of chips, chips formation, abrasion

## Abstract

In this study, the surface of new lapping films was analyzed, and the lapping finishing process was applied to RG7 tin bronze alloy. The research focused on examining lapping films with electrocorundum grains of nominal sizes 30, 12, and 9 μm, commonly used for achieving smooth surfaces. The manufacturing process involves placing abrasive grains and binder onto a polyester tape, resulting in a heterogeneous distribution of abrasive grains. The study investigates the impact of this random distribution on the performance of lapping films during material removal. Scanning electron microscopy was used to analyze the surface structure of abrasive films, revealing distinctive structures formed by the specific aggregation of abrasive grains. This study explores the influence of different nominal grain sizes on surface finish and aims to optimize lapping processes for diverse applications. The research also delves into microchip analysis, examining the products of the lapping film finishing process. Microchips were observed directly on the abrasive tool surface, revealing insights into their morphology and distribution. The chip segmentation frequency was determined, and they amounted to approximately 0.8 to 3 MHz; these are very high frequencies, which are unique for known chip-forming processes.

## 1. Introduction

**Figure 1:**
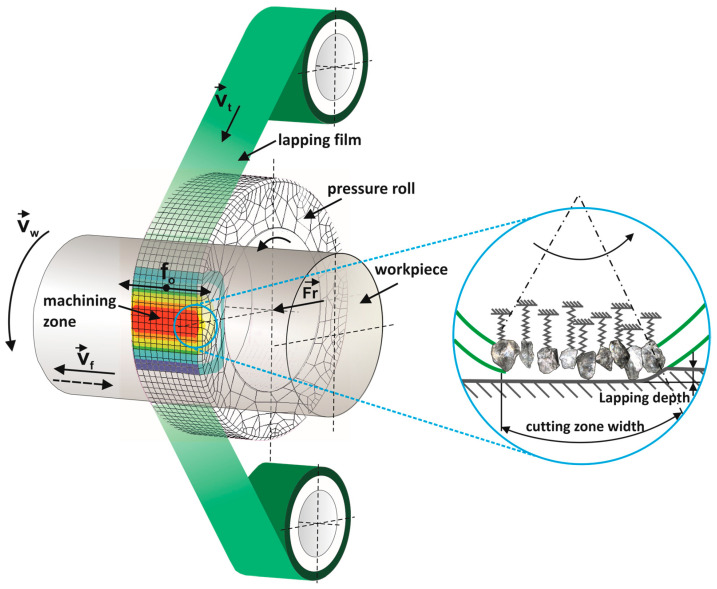
Kinematic diagram of rotary surface finishing using lapping films, where the following quantities are indicated on the diagram: v_t_—tool speed, v_w_—workpiece speed, v_f_—tool feed speed, f_o_—tool oscillation frequency, and F_r_—the pressure force of the pressing roller.

In microabrasive machining, such as precision grinding and microfinishing, the indentation of the tool into the workpiece material is significantly smaller than the radii of its corners and is comparable to the height of surface irregularities in the microcutting zone [1]. The variability of abrasive grain penetration into the workpiece material is an unfavorable, though inevitable, characteristic of microcutting processes. Microfinishing of technical surfaces with abrasive films is a modern method of finishing abrasive machining of external cylindrical and conical surfaces, as well as flat end faces [2]. It allows for highly efficient processing of surfaces of various materials, starting from metals, through plastics and composite materials, to ceramics and superhard materials [3]. The processing is carried out with precision abrasive films with a carrier made of thin polyester film covered with one or more layers of abrasive grains. The microfinishing process using abrasive films is characterized by a unique approach where the tool is used only once [4]. The abrasive film is unwound from a roll, and then pressed against the workpiece with a force Fr with a pressure roller (Figure 1). After use, the worn-out film is wound onto a roller, which simultaneously serves as a driving roller. This single-use feature distinguishes this method from processes utilizing endless [5] abrasive belts [6,7].

The article focuses on the investigation of a technology utilizing lapping film in the surface smoothing process, where this film moves at a velocity denoted as v_t_. A distinctive feature of this process is that the object undergoing treatment moves significantly faster at a velocity denoted as v_w_ than the tool used in the finishing process. To ensure maximum efficiency of the machining, it is beneficial to apply two additional motions, namely the oscillatory movement of the tool denoted as f_o_, and the longitudinal feed at a specified speed denoted as v_f_. The oscillatory movement of the tool, identified as f_o_, constitutes a key element introducing additional variability into the finishing process. This movement allows for the application of more intricate patterns of tool motion, potentially leading to more precise and controlled machining results. It can be customized to the specific requirements of the machining, enabling the achievement of diverse effects on the surface of the processed object. Moreover, the longitudinal feed at a specified speed v_f_ introduces a constant motion along the surface of the treated object. A characteristic feature of the finishing process is the application of pressure to the workpiece using a flexible roller, determining the nature of material removal [8,9]. It is worth noting that abrasive grains are pressed with the flexible roller, and at the same time, they are elastically embedded in a binder [10], making them susceptible to deformations and, ultimately, can be torn away from the binder under excessive forces [11]. The use of a flexible roller to apply pressure to the tool is a distinctive element of the surface finishing process. The flexibility of the roller allows for the adaptive interaction of the tool with the worked object, which is especially significant in the processing of freeform surfaces [12,13].

Flexible abrasive films allow for adjustments in the width of the cutting zone [14], providing the capability to control the width of the microfinishing zone [15], the size and distribution of pressure, and the number of cutting edges in the processing zone [16]. The width of the cutting zone directly affects various phenomena occurring in the processing zone [17]. The length of the zone depends on the diameter hardness, and working surface shape of the pressure roller, as well as on the workpiece being processed. The pressure distribution in the cutting zone influences the load on individual abrasive grains passing through the cutting zone [18], the depth of processing, the trajectory shape of abrasive grains, and consequently, the shape of microchips and processing efficiency [19]. A soft pressure roll (e.g., 50 Shore A) ensures a more uniform pressure distribution and a thinner layer of material being cut [20]. In such cases, grains work at more favorable angles of attack, directly affecting mechanical and thermal loads, as well as the quality of the top layer.

Research on micro and nanochips formed during the surface finishing process with abrasive films is unique on a global scale. The resulting products of the machining, especially in microcutting, including microdrilling [21], micromilling, and microturning, are very precisely examined. In micromachining, success is considered to be achieved with chip thicknesses below one micrometer [22]. The article presents chips with a thickness of approximately 100 nm, emphasizing the term ultra-precision machining. On a larger scale, chips were examined after machining processes: ultrasonic vibration-assisted cutting, turning, milling, and drilling [23]. The characteristic segmented structure of microchips has multiple physical causes in the processed material [24]. It has also been demonstrated that very high processing speeds also determine the segmented structure of the chips [25,26].

The main aim of this work was to analyze the surface of new lapping films and investigate the lapping finishing process applied to the CuSn7Zn4Pb6/RG7 tin bronze alloy. In the first phase, the study focused on the analysis of lapping films with electrocorundum grains of different sizes (30, 12, and 9 μm). This research aimed to understand the characteristics and performance of lapping films with distinct grain sizes, considering their production process and the resulting heterogeneous distribution of abrasive grains. The study delved into the impact of random grain distribution on the performance and efficacy of lapping films during material removal. It also explored the influence of nominal grain sizes on the overall surface finish achieved, providing insights for optimizing lapping processes across various applications. The research utilized scanning electron microscopy (SEM) to thoroughly analyze the surface structure of abrasive films, revealing characteristic structures and patterns formed by abrasive grains.

In the second phase, the investigation shifted to the analysis of microchips generated during the lapping film finishing process. The examination focused on understanding the morphology and distribution of microchips, offering valuable insights into their role in the finishing process. SEM images depicted the surface of lapping films after microfinishing, showcasing distinctive features and wear zones around abrasive grains. The study observed that microchips, products of the surface finishing process, were significantly smaller than the abrasive grains on the tool surface. The presence of wear zones was linked to concerns about tool-cutting efficiency and the potential for surface damage. The research highlighted the importance of lapping film surface characteristics in influencing chip behavior and distribution.

Moreover, the study observed the impact of different lapping films on tool wear, including the clogging and fracturing of abrasive grains. The use of a confocal microscope provided non-invasive insights into the microstructure of abrasive film surfaces, revealing details about the arrangement of abrasive grains in the binder structure. The investigation also touched upon the absence of spherical chips during the finishing of the RG7 bronze alloy, attributing it to the specific conditions of the process preventing material melting. The study presented detailed SEM images of microchips, their segmented structure, and variations in thickness, providing valuable information for understanding the material removal rate in the finishing process. Finally, the research extended to the analysis of processed surfaces using lapping films on the RG7 bronze alloy. Surface roughness parameters were determined, and the impact of different lapping films on surface characteristics was assessed. We concluded the study by presenting 3D layouts of machined surfaces after successive lapping film treatments and providing comprehensive data on surface roughness parameters.

In summary, the work aimed to comprehensively investigate the characteristics of lapping films, understand the behavior of abrasive grains and microchips during the finishing process, and assess the impact of different films on surface quality. The findings contribute to a better understanding of lapping processes and offer insights for optimizing these processes in various applications.

## 2. Materials and Methods

### 2.1. Surface Analysis of Lapping Films before the Finishing Process

To conduct the analysis of lapping film surfaces, a confocal microscope, Olympus LEXT OLS4000 (Tokyo, Japan), was used before the surface finishing process. Using a 100× magnification objective, it was possible to observe the sample surface in both confocal and optical modes. The measurement field was 128 × 128 μm. Three-dimensional surface measurements provide comprehensive information about the examined surface, moving away from contact measurements of surfaces [27,28]. Therefore, it was decided to conduct all surface measurements on the designated device. To analyze the surfaces of lapping films before the microfinishing process, a tabletop electron microscope, the Phenom ProX (Thermo Fisher Scientific Inc., Waltham, MA, USA), was also used.

### 2.2. Surface Analysis of Lapping Films and Microchips after the Finishing Process

To analyze the surfaces of lapping films after the microfinishing process and the worn tool surfaces, over 1000 SEM images of microchips, the products of microfinishing, were taken. This was conducted using the tabletop electron microscope PhenomWord Phenom ProX, equipped with an EDS spectrometer, which was used for chemical composition analyses of microchips. The electron microscope allowed for the examination of micro and nanochips at magnifications of up to 20 thousand times.

Using scanning microscope software, the thickness of chip segments and the width of chips perpendicular to their formation were measured. Measurements were performed for 50 chips, as products of lapping films with a specified grit size, totaling 150 measurements of chip width and an equal number of measurements of chip segment thickness. All results were presented in the form of Box plots, with the median of each dataset determined and the frame size set in the 25–75% range. To assess the variability of the obtained results, descriptive statistics were applied, including the calculation of the arithmetic mean, and determination of the minimum, maximum, and standard deviation.

### 2.3. Surface Finishing Process

Due to its good strength, durability, and resistance to wear, the processed material in the lapping film finishing process studies was Tin Bronze Alloy (CuSn7Zn4Pb6/RG7), which is used in the production of bearings, machine parts, and sliding elements. These applications require very precise finishing, which abrasive films can provide. The processing conditions are presented in Table 1.

The GW-1 microfinishing attachment was utilized in the investigation of the machining process. The GW-1 type microfinishing attachment is designed to be affixed to an engine lathe in the cutter holder seat. This enables the interchangeable use of tools with widths of 1/2″, 1″, or 2″. The tool feed rate is v_f_ = 0…90 (max. 500) mm/min, the oscillation frequency is f_0_ = 0…500 1/min, and the oscillation amplitude is A = 2.5 mm. The range of the roll’s pressure forces is Fr = 10…90 (max. 200) N, and it was achieved using a pneumatic actuator supplied from a system with a pressure of 0.6 MPa. The input voltage was 230 V, the installed power was 400 W, the overall dimensions were 575 × 250 × 300 mm, and the mass was approximately 25 kg.

### 2.4. Surface Analysis after the Surface Smoothing Process

The Olympus LEXT OLS4000 microscope was utilized for the analysis of the machined workpiece surface, employing a 100× magnification objective. The measurement field covered an area of 128 × 128 μm.

From all 27 measured surfaces within the range of 128 × 128 μm, the following surface roughness parameters were determined (according to ISO 25178 [29]) for description:

- Sp—surface peak height;
- Sv—surface valley depth;
- Sz—surface height;
- Sa—arithmetic mean height.

To examine the variability of the results, descriptive statistics were applied for all roughness parameters, including the arithmetic mean, minimum, maximum, and standard deviation.

To determine the width of the machining track, the analysis of segmentation patterns was applied to the surface roughness profile, which is generated perpendicular to the direction of the machining tracks. The method for determining patterns employs the Watershed Segmentation method according to ISO 16610-45 standard [30]. The Watershed Algorithm is employed to identify boundaries between areas of varying heights on the surface roughness profile. As a result, collective information about the surface structure is obtained, and segmentation areas are determined based on natural divisions. The method identifies motifs consisting of sequences of triplets: peak–valley–peak. These motifs identify tool marks on the finished surface and enable the determination of the height and width of the machining signatures. Roughness profiles were extracted from all 27 surfaces. Subsequently, all surface roughness profiles underwent motif analysis. To examine the variability of the obtained results, descriptive statistics of the AR parameter were determined for all analyzed surface roughness profiles. The AR parameters represent the mean spacing of roughness motifs directly related to the width of machining tracks.

## 3. Results and Discussion

The presented study involved the analysis of the surface of new lapping films. Subsequently, the lapping finishing process was applied to RG7 tin bronze alloy. In the second phase of the study, the focus was on a detailed analysis of microchips, which constituted the products of the processing within this procedure. At the conclusion of this chapter, the results of the surface analysis subjected to the treatment are presented.

### 3.1. Studies of Lapping Films

In this research, we delve into the examination of lapping films featuring electrocorundum grains with nominal sizes of 30, 12, and 9 μm. In the subsequent sections of the article, abbreviations are employed to denote specific grades of abrasive films: 30LF (lapping film with a nominal abrasive grain size of 30 μm), 12LF (lapping film with a nominal abrasive grain size of 12 μm), and 9LF (lapping film with a nominal abrasive grain size of 9 μm). These particular gradations are widely employed to attain surfaces of remarkable smoothness, making them of significant interest for various applications. The investigation centers on understanding the characteristics and performance of lapping films across these distinct grain sizes.

**Figure 2:**
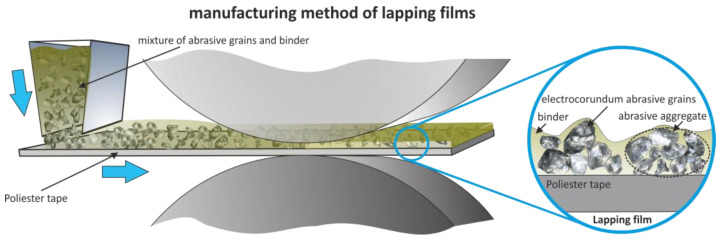
Schematic depicting the production of lapping film.

The production process of lapping films unfolds with the placement of a mixture comprising abrasive grains and binder onto a polyester tape. Subsequently, the film undergoes a shaping process and is subjected to drying (Figure 2). Throughout this manufacturing journey, abrasive grains find their place on the tape in a stochastic manner, creating a heterogeneous distribution [2]. To ensure cohesion, these grains are delicately enveloped with a thin layer of adhesive.

The randomness in the distribution of abrasive grains on the tape introduces an element of complexity and variability to the resulting lapping films. This study aims to unravel the implications of such random distribution, shedding light on how it influences the performance and efficacy of lapping films during the material removal process. Additionally, it explores the impact of different nominal grain sizes on the overall surface finish achieved, providing insights into the optimization of lapping processes for diverse applications.

**Figure 3:**
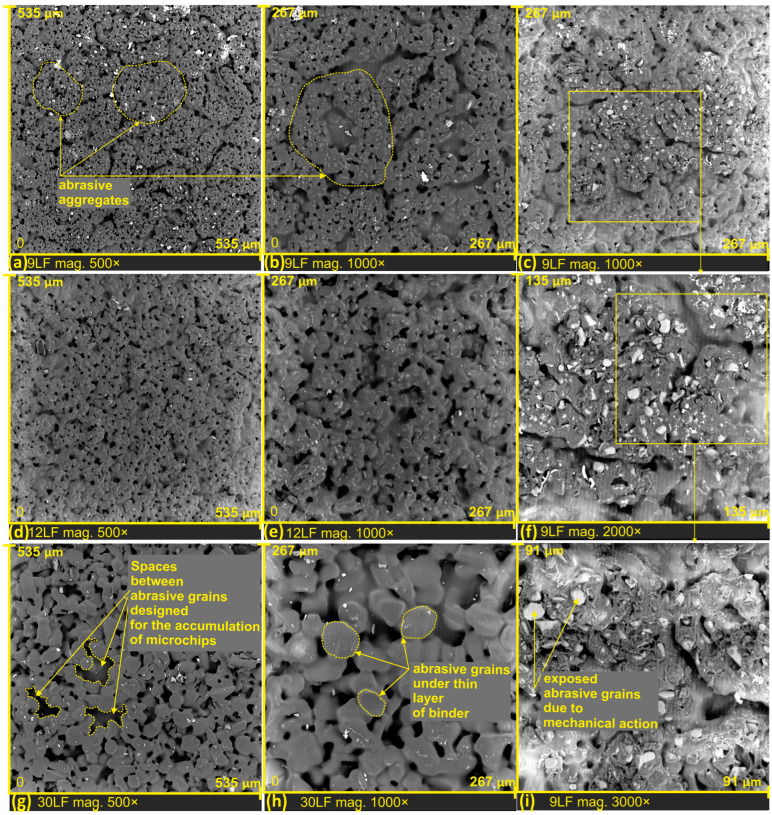
SEM images of new lapping films, with a nominal size of 9 µm (**a**,**b**), 12 µm (**d**,**e**), 30 µm (**g**,**h**); SEM images of 9LF films with mechanically removed thin binder layer and exposed abrasive grains of alumina (**c**,**f**,**i**).

During the examination of the surface structure of abrasive films, a scanning electron microscope was employed. The obtained results, presented in the form of SEM images, are depicted in Figure 3. All analyzed films were photographed at two different magnifications: 500× and 1000×, enabling a detailed comparative analysis of their structure.

Scanning electron microscopy allows for a thorough microscopic analysis of the surface structure of abrasive films. Images captured with a scanning electron microscope (SEM) are particularly valuable for visualizing microstructure, and topography, as well as detecting potential defects or damage on the surface of the films.

In Figure 3a,b, a lapping film with a nominal grain size of 9 μm is presented. Through the example of this specific film, we can observe a phenomenon of specific aggregation of abrasive grains, leading to the formation of characteristic structures. The production process of abrasive films significantly influences the shaping of abrasive aggregates on their surface, surrounded by empty spaces. These empty spaces effectively serve for transporting and removing processed products from the microfinishing zone (Figure 3g). It is worth noting that the distinctive structures visible in Figure 3 may play a crucial role in the efficiency of the grinding process, the transport of processed products, and the overall quality of the obtained surface. Further research and analysis of this microstructure can contribute to a better understanding of the impact of abrasive grain structure on the performance of abrasive films in various applications.

In the SEM images (Figure 3c,f,i), the surface of the 9LF abrasive film is depicted after the mechanical removal of the binder layer. Owing to this procedure, we can precisely observe the distribution of abrasive grains within the binder and comprehend the structure of the abrasive tool. The process of mechanically removing the binder layer allows us to look beneath the surface, revealing microscopic details regarding the distribution of abrasive grains and the overall structure of the tool. SEM image analysis facilitates a thorough understanding of how abrasive grains are embedded in the binder, which is crucial for characterizing the abrasive tool and its potential machining properties. After removing the upper binder layer, abrasive grains were exposed, confirming that the abrasive grains were fully embedded in the tool binder. The abrasive grains are randomly distributed on the tool’s surface and are not specifically arranged on it. This is directly attributed to the production method of lapping films, where the mixture of binder and abrasive grains is spread on the surface of a polyester film through a rubbing process.

**Figure 4:**
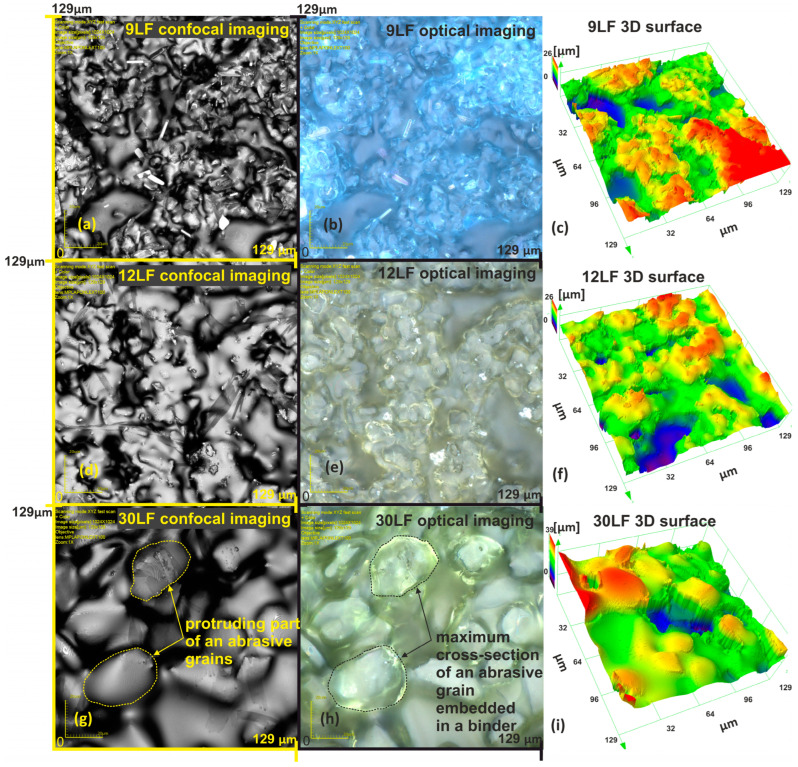
Images of new lapping films post data acquisition with the Olympus OLS4000 microscope: confocal imaging: (**a**) 9LF, (**d**) 12LF, (**g**) 30LF; optical imaging: (**b**) 9LF, (**e**) 12LF, (**h**) 30LF; 3D surface height map: (**c**) 9LF, (**f**) 12LF, (**i**) 30LF.

The method of mechanical binder removal is a destructive process; therefore, for further investigations into the topography of the abrasive film surface, a confocal microscope was employed. This microscope operates in two modes, with the first being the confocal mode (Figure 4a,d,g), enabling the acquisition of data about the examined surface in a 3D arrangement (Figure 4c,f,i). These images confirm the presence of indentations around aggregated grains, which may serve as potential storage locations for machining by-products. In comparison to the mechanical binder removal method, the confocal microscope offers a non-invasive alternative, allowing for precise examination of the microstructure of the abrasive film surface. The second mode of data acquisition, namely the optical mode, by the confocal microscope, is highly useful in the examination of lapping film surfaces. Due to the fact that abrasive grains are situated beneath a thin layer of transparent binder, we can observe the arrangement of abrasive grains in the binder structure. Additionally, we can determine complete cross-sections of abrasive grains even though they are submerged. Figure 4g shows a portion of a grain protruding above other grains, while the optical in Figure 4h allows for the determination of the actual size of the grain. Using the analysis of optical images from a confocal microscope, the number of abrasive grains per square millimeter on the film surface was determined: for 30LF, the number of abrasive grains is 1200; for 12LF, the number is 5760; and for 9LF, the quantity is 10,200 abrasive grains per square millimeter of the abrasive film.

### 3.2. Research on Microchips, Lapping Film Finishing Products

**Figure 5:**
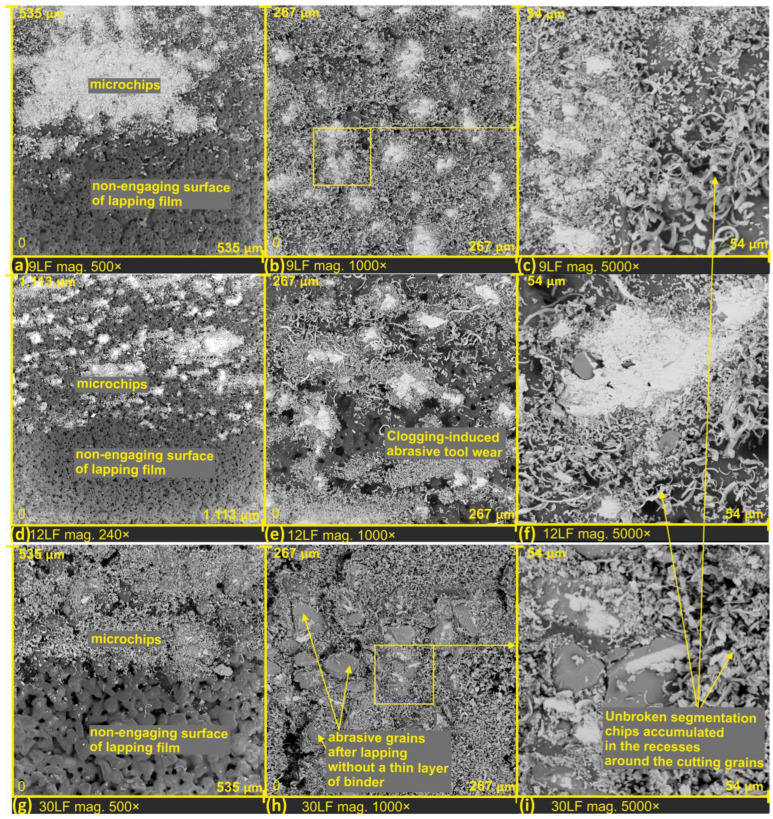
SEM images of lapping films after microfinishing, with a nominal size of 9 µm (**a**–**c**), 12 µm (**d**–**f**), 30 µm (**g**–**i**).

**Figure 6:**
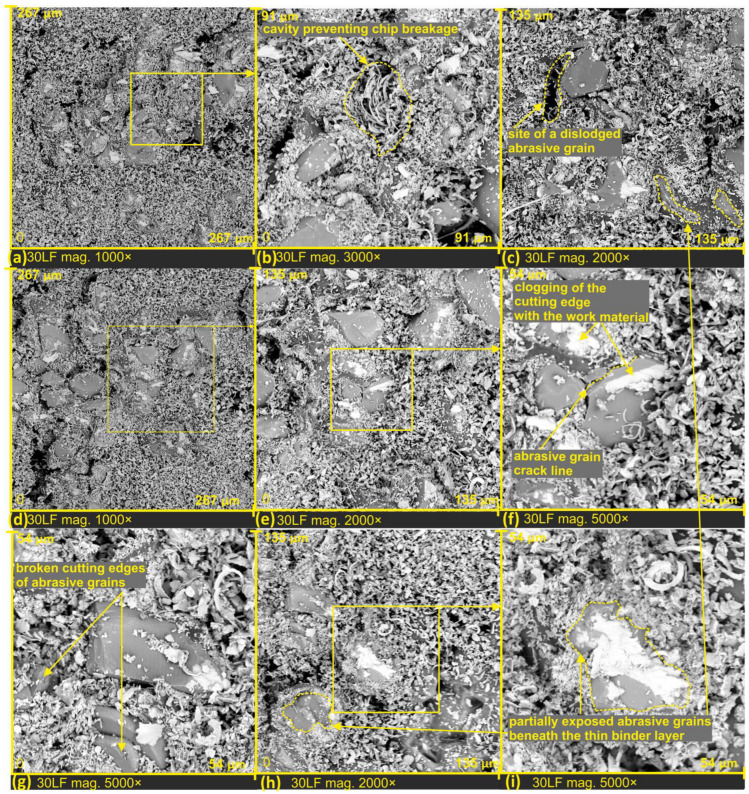
SEM images of lapping films after microfinishing, with a nominal size of 30 µm (**a**–**i**).

**Figure 7:**
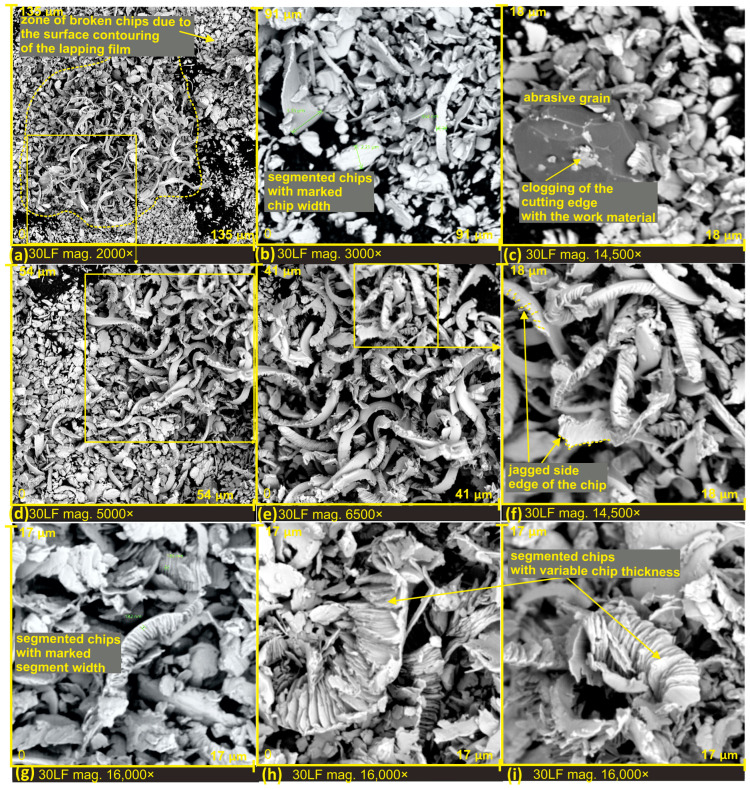
SEM images of microchips formed after lapping film processing with a nominal grain size of 30 µm (**a**–**i**).

**Figure 8:**
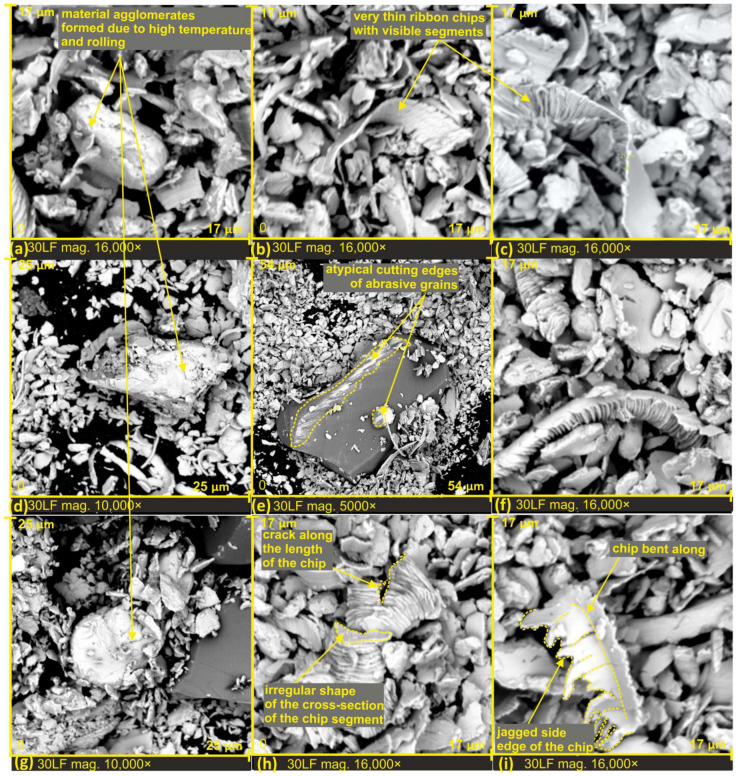
SEM images of microchips formed after lapping film processing with a nominal grain size of 30 µm (**a**–**i**).

**Figure 9:**
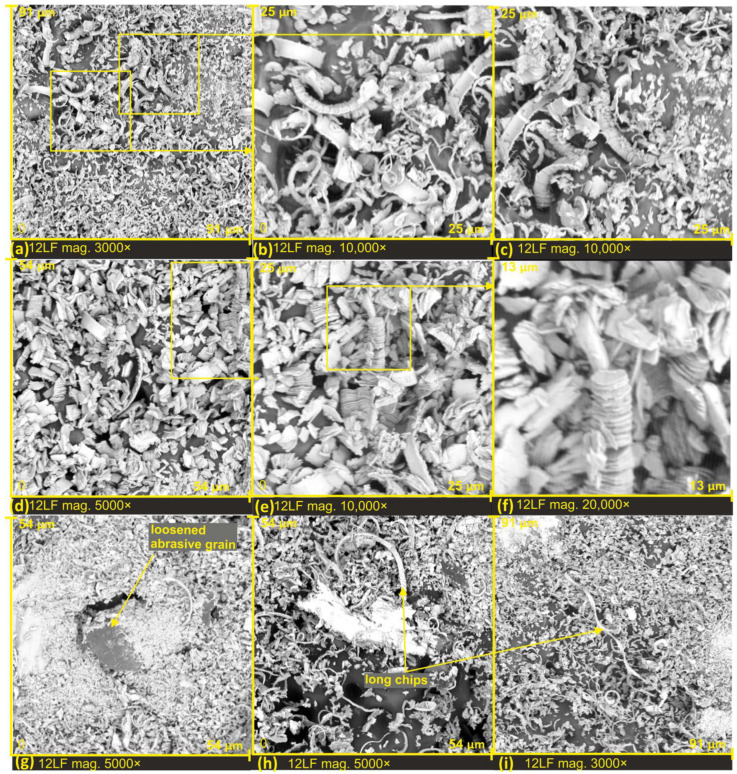
SEM images of lapping films after microfinishing, with a nominal size of 12 µm (**a**–**i**).

**Figure 10:**
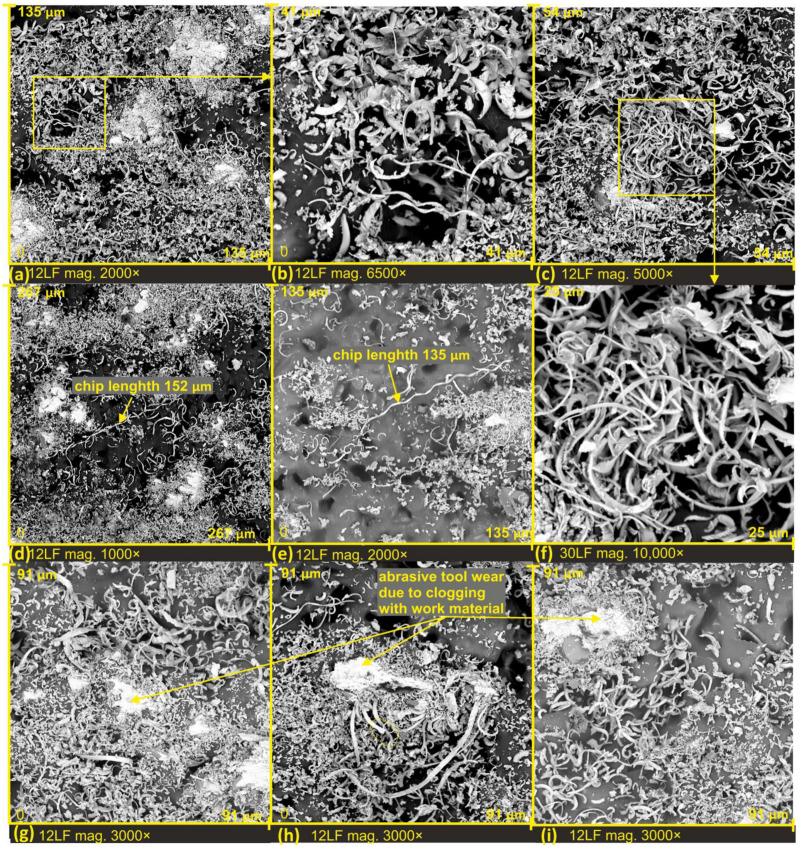
SEM images of lapping films after microfinishing, with a nominal size of 12 µm (**a**–**i**).

**Figure 11:**
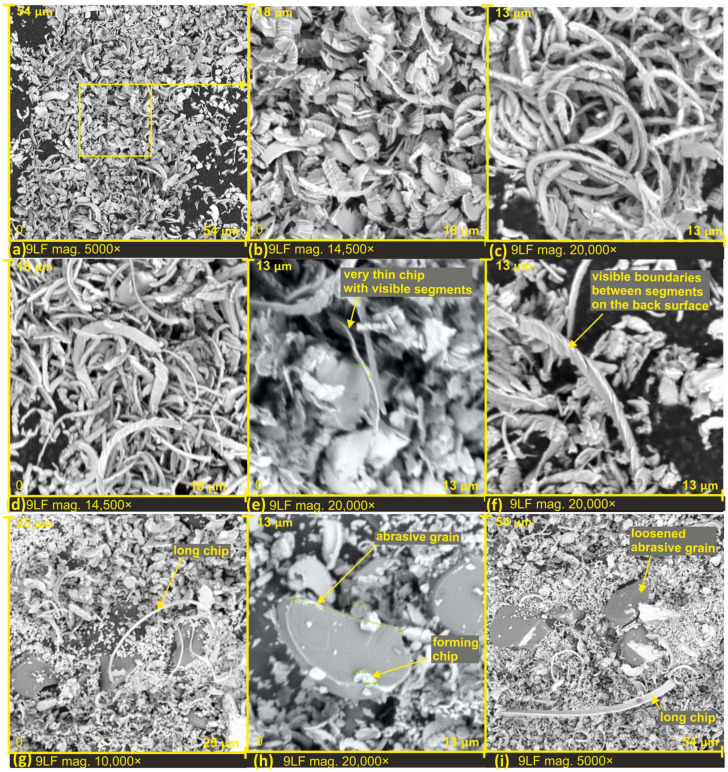
SEM images of lapping films after finishing, with a nominal size of 9 µm (**a**–**i**).

In the investigation of microchips generated during the lapping film finishing process, examinations were conducted directly on the abrasive tool surface after the completion of the process (see Figure 5, Figure 6 and Figures 9–11). Due to the non-conductive nature of the abrasive tool surface, which is crucial for obtaining sharp images at high magnifications during electron microscope measurements, and considering that the chips are of micro and nanometric dimensions, graphs of the microchips at high magnifications (see Figure 7 and Figure 8) were captured on the double-sided carbon tape surface. On this adhesive surface, the chips were placed by pressing them onto the worn surface of the lapping films, as the tool surface itself was not conducive to such imaging requirements. This approach allowed for the precise examination and visualization of the micro and nanoscale features of the microchips, providing valuable insights into their morphology and distribution for a comprehensive understanding of the lapping film finishing process.

Figure 5 presents SEM images of the lapping film surfaces on the edge of the machined zone (Figure 5a,d,g). This allows us to observe two distinct zones in the images: the zone not involved in the processing (lower half of the image) and the tool zone after the processing, with visible filled spaces around the abrasive grains. In SEM images, the chips appear brighter compared to the bonding layer on the abrasive tool.

The examination was conducted on three grades of lapping films with abrasive grain sizes of nominal values: 9 μm (designated as 9LF, see Figure 5a–c), 12 μm (12LF, see Figure 5d–f), and 30 μm (30LF, see Figure 5g–i). Specifically, the SEM images provide insights into the distinctive characteristics of the lapping film surfaces at different grain sizes, highlighting variations in the machined zones and the tool surfaces after the processing. The filled spaces around the abrasive grain chips are evident, offering valuable information for understanding the effects of different lapping film grades on the resulting surface morphology.

A distinctive feature is that microchips, as products of the surface smoothing process, are significantly smaller in size compared to the abrasive grains on the tool surface. Upon examining SEM images, it was observed that the surface topography of the lapping film plays a crucial role in the storage and removal of processed products beyond the machining zone. On the surface of the 30LF lapping film, no wear zones around the highest abrasive aggregates were observed (see Figure 5h). Conversely, wear zones around the highest peaks were noticed on the 12LF films (see Figure 5f) and 9LF films (see Figure 5c), indicating a potential weakening of the cutting capabilities of the tool and the possibility of damaging the machined surface. Several factors contribute to the impact of wear zones on tool performance and surface quality: reduction in abrasive effectiveness, increased friction and heat generation, altered surface finish, and process stability and predictability.

These findings underscore the importance of lapping film surface characteristics in influencing the behavior and distribution of chips during and after the finishing process. The absence of wear zones on the 30LF film suggests a more efficient evacuation of microchips, potentially contributing to a more effective and controlled finishing process. In contrast, the presence of wear zones on the 12LF and 9LF films raises concerns about tool-cutting efficiency and the potential for surface damage. This insight provides valuable guidance for optimizing lapping film selection based on the desired surface finish and the specific requirements of the machining process. Based on these investigations, it can be concluded that the speed of lapping film movement should be adjusted individually for each machining operation. This is because the optimal filling of the space with processed products depends on the speed of lapping film movement. Importantly, the overfilling of this space should be avoided, as it may lead to negative consequences.

Observations revealed tool wear in the form of clogging induced around the highest abrasive aggregates, with a zone of fractured chips surrounding this area. The chips in this zone are short and densely arranged, while those farther away exhibit a greater depth of tool surface and longer ribbon-like chips that remain unfragmented (see Figure 5c,f). Consequently, it can be inferred that during the surface finishing process, there is no formation of discrete chips, characterized as small, discontinuous chips. Instead, longer chips are generated, and these undergo breakage as they are carried out of the machining zone due to compression forces. This observation implies that the microfinishing process does not produce individual element chips but rather longer chips prone to breakage when lifted from the machining zone. The presence of fractured chips and the differences in their characteristics based on their location suggests dynamic variations in the micropolishing process.

Based on the analysis of the examined tool surfaces, it was observed that just before the micromachining process, the abrasive grains are exposed from beneath a thin binder layer, and only then does the cutting occur (Figure 5h,i and Figure 6c,h,i).

Unfavorable phenomena were observed on the surface of the lapping film, directly influencing the reduction in the quality of the machined surfaces. As a result of the impact of large forces in the machining zone, abrasive grain fracture (see Figure 6f), and dislodging from the lapping film surface (see Figure 6c). Unfortunately, this can have a detrimental effect on the quality of the machined surface, leading to the formation of individual deep scratches due to the unrestricted movement of loose abrasive in the machining zone. Loose abrasive in the cutting zone, where high forces prevail, can cause many problems. This phenomenon is utilized in water jet machining, where loose abrasive is ejected into the medium under high pressure, or sometimes the pressure of the medium alone is sufficient to induce erosive action on the surface [31]. Additionally, chipping of grain peaks was observed (see Figure 6g) along with the clogging of the cutting edge with work material (see Figure 6f).

In order to observe the microchip structure at high magnification as a product of surface finishing, the processed products were placed on adhesive carbon tape by pressing it onto the lapping film. This process allowed the chips to be extracted from the inter-grit space of the abrasive tool. In SEM images shown in Figure 7a,d, the difference in how chips are stored on the film surface is clearly visible. In Figure 7a, the dashed line marks the area corresponding to the space between grains on a substantially deep film, resulting in microchips not undergoing as much fragmentation as the surrounding chips. There are zones with completely fractured chips, densely packed, and chips from deep tool zones where they could be stored without damage (see Figure 7e). This phenomenon enables the investigation of the structure and morphology of these chips. In Figure 7c, a dislodged abrasive grain from the tool surface is evident. It can be confidently asserted that the grain was torn from the lapping film surface during the cutting process, as clogging of the cutting edge with work material is observable. The grain size is approximately 10 μm, and such a freely moving grain in the machining zone can lead to the formation of very deep scratches on the smoothed surface. Upon studying the morphology of chips generated in the surface finishing process, it can be confidently stated that all resulting chips have a segmented structure, which has a direct connection with a high material separation speed [32].

However, due to the highly varied shape of electrocorundum grains, and consequently their cutting edges, the formed chips exhibit significant diversity. One can observe chips with jagged edges (Figure 7f), segmented chips with variable thickness (Figure 7h,i), and chips of different widths and shapes.

The thickness of the chips varies significantly, resulting from the diverse rake angles of the grains. Chips with a thickness of at least 20 times greater than that of a single segment of a chip can be observed (see Figure 8c). Additionally, there are very thin chips with a thickness equivalent to a single chip segment (approximately 100 nanometers) (see Figure 8b,c). It is worth noting that these extremely thin chips still exhibit a segmented structure; only the inclination angle of the segments changes, directly depending on the rake angle of the grains.

The variation in the shape of the cutting edges of the grains can also be observed by analyzing the irregular shape of the cross-section of the chip segments (see Figure 8h) and the change in cutting depth and cracks along the length of the chip. This observation further confirms the presence of a highly asymmetrically bent chip, with one side featuring a very jagged edge. On this chip, perpendicular discontinuities can also be observed, forming at the junction of segments (see Figure 8i). The atypical shape of the cutting edge of the abrasive grain can be observed, for instance, in Figure 8e, where the grain possesses two cutting edges. The first one is narrow and long, while the second one arises from the peculiar shape of the electrocorundum.

Unusual agglomerates among the finishing products were also observed, resulting from the influence of high temperatures and the rolling of chips that fused together to form a larger rolled structure (see Figure 8a,d,g).

Segmented chip structures were also observed on the finishing products of lapping films with a nominal grain size of 12 μm (see Figure 9a–f). All chips exhibit a segmented structure with diverse shapes and thicknesses, clearly visible at high magnification (see Figure 9f), where individual chip segments are connected only in a narrow portion on the side facing the cutting edge of the abrasive grain.

Additionally, one can observe the dislodging or loosening of abrasive grains after the cutting process (Figure 9g). In this case, the abrasive grain was lifted back to its position from the finishing zone, but its loosening could still have had adverse effects on the machined surface.

Under exceptional conditions, where the chip has not been fractured by pressing it through the abrasive film onto the machined object, we can observe the formation of long chips in the lapping film finishing process (see Figure 9h,i and Figure 10h) as well as in Figure 10d, where the chip’s length measures 152 μm, and Figure 10e, showing a chip with a length of 135 μm. Long chips accumulated in the recesses of the recesses of the film, can also be observed in Figure 10a–c,f, with the most interesting being Figure 10b, where the chips form a kind of bridging structure.

However, in Figure 10g–i, small wear zones around the cutting edges of the chips can be observed. It is noteworthy that the zone with very fragmented chips around the edge is limited in this case, and the chips gather in a characteristic ribbon-like form. This suggests that there is an optimal space for the accumulation of machining products.

In Figure 11, the processing products on the surface of the film with a nominal grain size of 9 μm are presented.

In this case, all chips also exhibit a segmented structure (see Figure 11a–d), with varying thicknesses and diverse shapes of the segments. The occurrence of chips with a thickness equivalent to a single segment, namely 99 nanometers, was also observed (see Figure 11e). Characteristic perpendicular discontinuities to the length of the chip, present at the junctions of segments on the side facing the cutting edge, were noticed on the chip in Figure 11f. Similarly to the 30LF and 12LF films, the presence of long chips was observed on the 9LF film (see Figure 11g,i).

In Figure 11h, the proportion between the maximum width of the abrasive grain and the width of the chip is clearly visible. The width of the grain is 6.53 μm, while the width of the chip is 0.670 μm. In this specific case, it means that the chip is approximately 10 times narrower than the total width of the abrasive grain.

**Figure 12:**
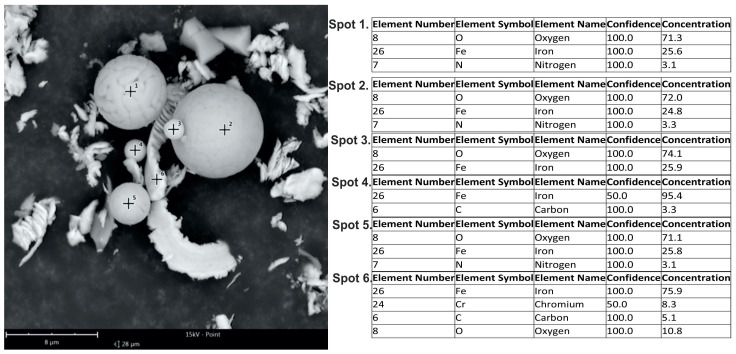
SEM images of microspheres after microfinishing of tool steel heat-treated to 60 HRC along with the analysis of their chemical composition.

After extensive examinations of machining products following the lapping films finishing process, and after conducting and analyzing over a thousand SEM images, no occurrence of spherical chips, or microspheres, was observed. The only atypical products are chunks of material formed due to the influence of temperature and rolling (see Figure 8a,d,g). It can be confidently stated that during the smoothing of the RG7 bronze alloy, material melting does not occur, leading to the absence of spherical chip formation. Therefore, the conclusion is drawn that temperatures in the cutting zone did not exceed 1040–1080 degrees Celsius, which is the melting temperature of bronze. This phenomenon is not common because, during the finishing of the workpiece made from tool steel when it was heat-treated to 60 HRC, the presence of microspheres, or spherical chips, was observed in a maximum size of 8 μm. Their chemical compositions, in the vast majority, are iron oxides (Figure 12).

**Figure 13:**
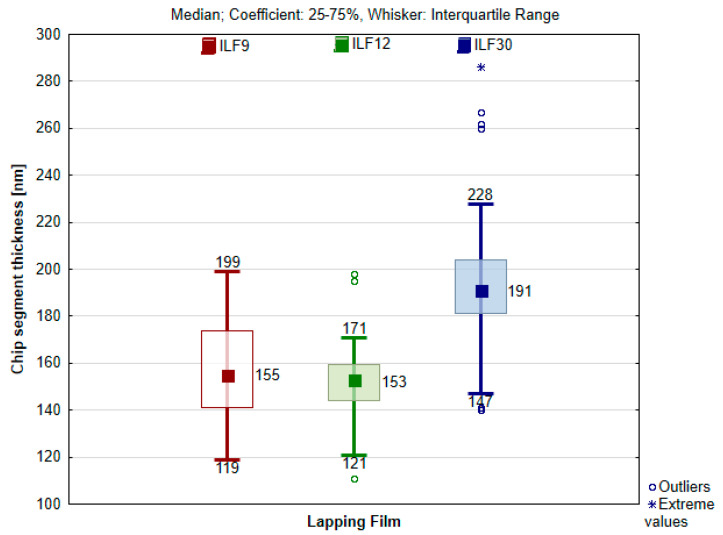
Box plot of chip segment thickness depending on the nominal size of abrasive grain on lapping film.

To determine the material removal rate in the surface finishing process using lapping film [33], the thickness of the chip segment was measured based on SEM images, utilizing the electron microscope software. Figure 7g illustrates a sample chip with the determined segment thickness. The measurement results are presented in Figure 13, and descriptive statistics of the obtained results are compiled in Table 2. The measurements were conducted for three grades of lapping films with nominal grain sizes of 9, 12, and 30 μm.

There was no significant difference observed in the thickness of chip segments, the products of lapping film processing with nominal grain sizes of 9 and 12 μm. The difference in the average thickness of segments is about 4 nanometers. Interestingly, the thinnest segments are formed during the process with the 12LF film, which also has the smallest standard deviation, measuring only 17 μm. On the other hand, the thickest average segment thickness was obtained as a result of the interaction with the 30LF film, where the thickness is just under 200 nanometers, about 40 nanometers more than with the 9 and 12LF films. Considering that all blades were cutting at the same speed, namely 105 m/min, it means that the thickness of the segment depends not only on the cutting speed but also on the cutting depth. In the case of 30-micrometer grain size, the cutting depth is greater for obvious reasons.

The determined average chip segment thickness allows for the calculation of the chip segmentation frequency *f_s_* (1) [34] as the ratio of the cutting speed *v_w_* to the distance that a grain must traverse in the cutting direction to separate a single chip segment. This distance is expressed as the product of chip segmentation length *ls* and the sine of the tangential grain cutting angle *φ*. The tangential grain cutting angle in microcutting of highly ductile materials typically falls within the range of 5 to 15° [35] (Table 3). Frequencies within this range are also determined.

Chip segmentation frequency:

(1) $$f_{s}=\frac{Vw}{\frac{ls}{\sin\varphi }}$$

The highest chip segmentation frequency was determined for the 12LF lapping film, directly linked to the average smallest segment thickness. It is worth noting that in traditional machining methods, such as turning, material separation frequency is expressed in kHz [36]; in publication [37], the orthogonal cutting process was analyzed, and a chip separation frequency of 9.66 kHz was obtained at a tool speed of 90 m/min. The thickness of separated segments is in the micrometer range. The specificity of surface finishing using lapping films lies in the fact that the thickness of the chip segment is expressed in nanometers, resulting in enormous material separation frequencies at the megahertz level [38,39,40,41]. Chip segmentations arise during high-speed machining due to the formation of adiabatic shear bands [42,43]. The segmented structure of chips is a desirable phenomenon because, owing to this specific construction, chips can more tightly fill the spaces between grains through easier chip breaking.

**Figure 14:**
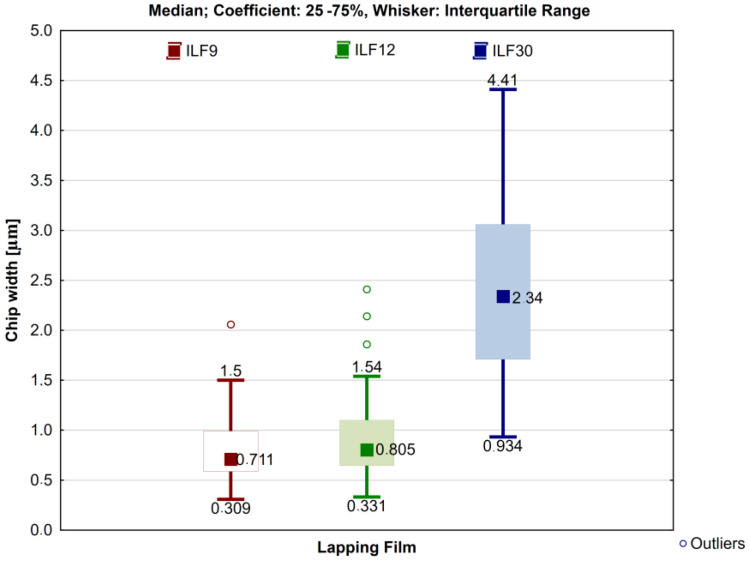
Box plot of chip width depending on the nominal size of abrasive grain on lapping film.

To attempt to estimate the width of cutting traces, the width of the chips was determined using scanning microscope software. Sample results are presented in Figure 7b. The obtained chip widths, depending on the type of lapping film used in the microcutting process, are shown in Figure 14, while descriptive statistics are compiled in Table 4.

The average chip width for the surface finishing process using lapping films 12LF and 9LF shows a slight difference. In the case of the 12LF film, the chip width is only approximately 70 nanometers wider, measuring precisely 889 nanometers. This means that the chip width for the 12LF film is 13.5 times smaller than the nominal abrasive grain size, while for the 9LF film, this ratio is 11. The highest average chip width was obtained for the 30LF film, reaching 2.42 μm, with a ratio of the nominal grain size to the average chip width being 12.35 times. The obtained values vividly illustrate the minimal involvement of an abrasive grain fragment in the material removal process. In general, it can be concluded that the width of the cutting zone in the case of lapping films is less than one-tenth of the nominal abrasive grain size (Figure 11h).

### 3.3. Analysis of Processed Surfaces Using Lapping Films

**Figure 15:**
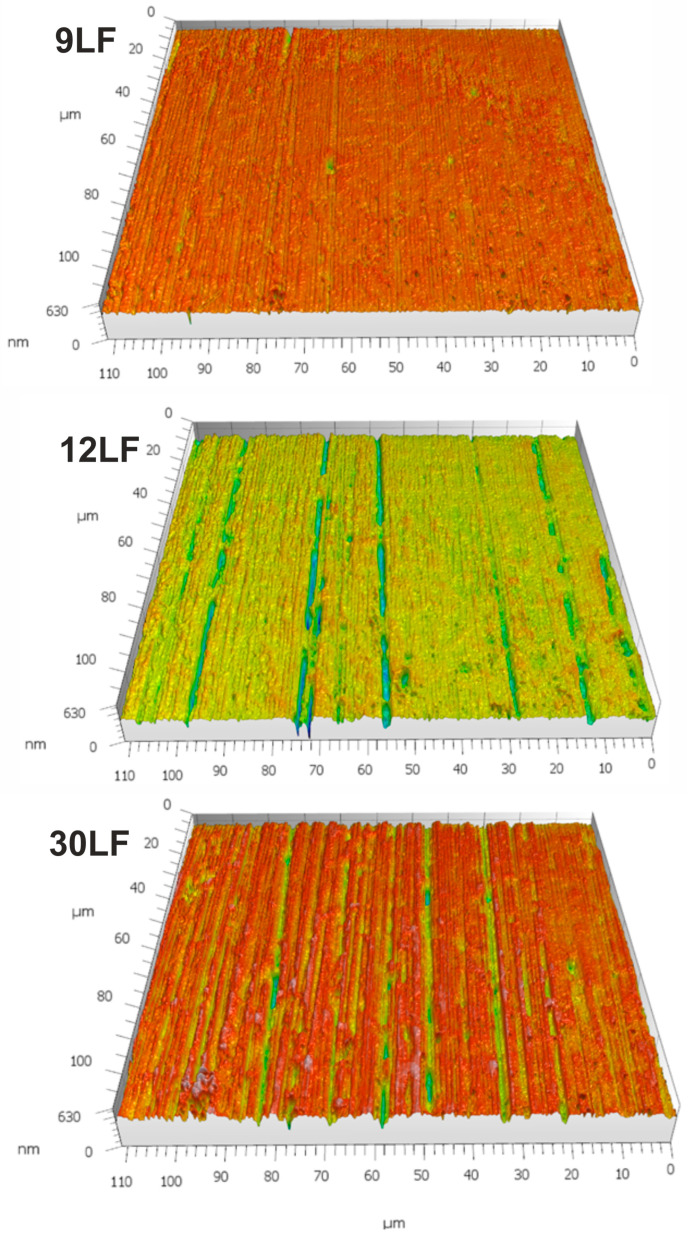
Machined surfaces, presented in a 3D layout after treatment with 9LF, 12LF, and 30LF lapping films.

To investigate the outcomes of lapping film processing, the treated surfaces were examined. The workpiece is made of a bronze alloy labeled as GR7. The processing, in accordance with industry practices, was carried out sequentially. In the initial operation, the surface was finished using a 30LF film, with a finishing time of 60 s. Subsequently, surface roughness measurements were taken at nine locations, and all analyses were conducted on the collected dataset. Next, the workpiece surface was finished with a 12LF film for an additional 60 s, followed by measurements at nine locations. Finally, a 9LF film was applied, and the geometric structure of the workpiece was measured at nine locations. Figure 15 illustrates sample surfaces after successive lapping film treatments. The most pronounced machining traces were observed on the surface treated with the 30LF film. The impact of the 12LF film is quite characteristic, as it is evident that some traces from the 30LF film were removed but not all. Only the final operation eliminated all traces from previous treatments.

The use of parameters for assessing surface roughness calculated from a 3D surface, not just from the surface profile, allows for more accurate conclusions about the roughness of the examined surfaces [44]. Table 5 presents a comprehensive compilation of all the determined parameters along with their corresponding descriptive statistics.

As expected, following each application of lapping film and abrasive grain reduction, the Sa parameter decreased. After processing with the 30LF film, it measured 26 nanometers, while after treatment with a film featuring nominal grain size, it decreased by half to 13 nanometers. Additionally, in line with expectations, as the grain size reduced on the film surface, the maximum peak height on the surface also decreased. After processing with the 30LF film, it was 0.19 μm, and after smoothing with the 9LF film, it decreased almost by half to 0.10 μm.

Regarding the depth of valleys on the processed surface, after smoothing with the 30LF film, it was 0.70 μm. After smoothing with the 12LF film, the depth decreased to 0.56 μm, and then, after further finishing with the 9LF film, it increased again to 0.67 μm. This is not a surprising phenomenon, as we must consider that this parameter specifies the maximum depth of the valley. Therefore, a single deep scratch is enough to dramatically increase the value of this parameter. Upon analyzing the results of the finishing process, it can be speculated that the phenomenon of grain detachment from the film surface may be responsible for the formation of individual deep scratches. Consequently, the application of parameters sensitive to this phenomenon may not fully represent the true nature of the phenomenon.

**Figure 16:**
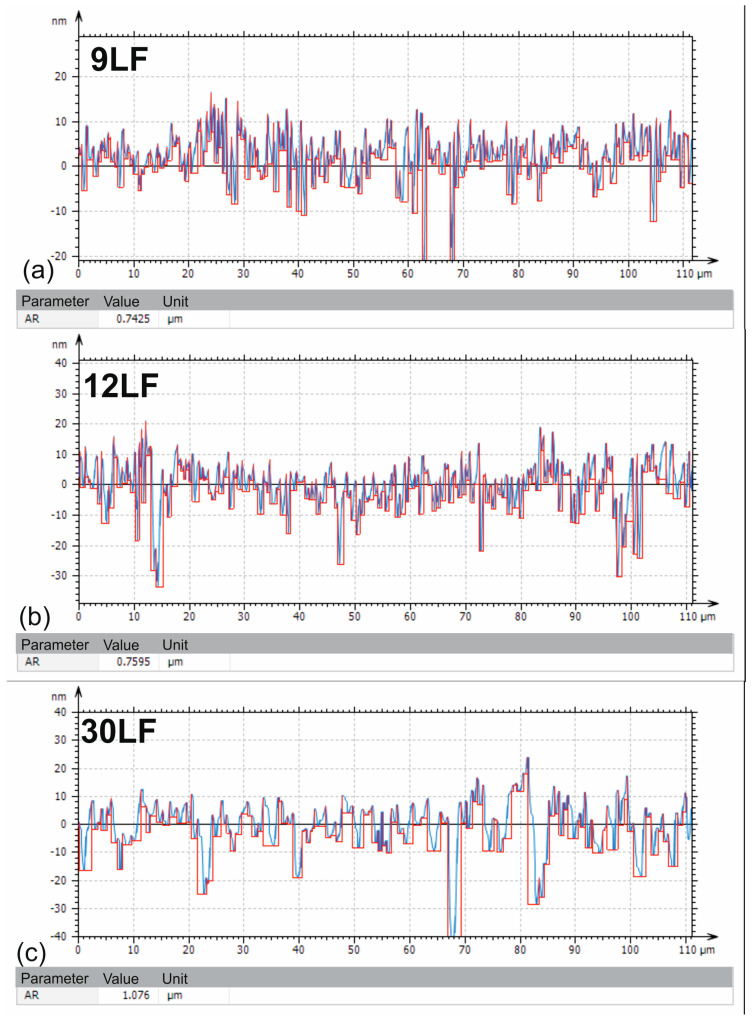
The surface profile after processing with films, (**a**) 30LF, (**b**) 12LF, (**c**) 9LF, underwent analysis using the Watershed Segmentation method (red line), following ISO 16610-45 standard.

In the preceding subsection of the article, the average chip widths, and products of the lapping film processing, were determined. Due to their segmented structure, it can be assumed that the chip width is nearly equal to the cutting track width. It was decided to find a parameter that would describe the cutting width, i.e., the width of the machining track on the surface treated with abrasive films. The proposed approach involves utilizing the segmentation patterns analysis determined on the surface roughness profile, which is generated perpendicular to the direction of machining tracks. Sample profiles (in blue) with overlaid motifs (in red) are presented in Figure 16. The values of the determined AR parameters, representing the mean spacing of roughness motifs directly related to the width of machining tracks, were compiled in Table 6 along with descriptive statistics for all analyzed surface roughness profiles.

The average width of the machining track on the surface after finishing with the 30LF film is approximately 1.2 μm. The average chip width, a product of the processing with the 30LF film, is about 2.4 μm. Hence, it can be concluded that due to the crossing of tracks and the overlay of successive machining tracks during the finishing process, the width of the machining track on the machined surface decreases by half. Interestingly, in the case of the 12LF and 9LF films, the average chip width was about 0.8 μm, while the width of the machining track on the machined surface, in both cases, is approximately 200 nanometers wider. This may suggest the presence of machining tracks on the surface after previous treatments. Consider that the efficiency of material processing is influenced not only by external factors. The authors [45] of the article have illustrated that comprehending the interplay between meso-scale and macro-scale structures is essential to enhance the effectiveness of abrasive films in surface smoothing applications.

## 4. Summary and Conclusions

The presented study focused on the analysis of lapping films and their application in the finishing process of RG7 tin bronze alloy. The investigation included a detailed examination of lapping film microstructure, emphasizing the impact of random grain distribution on film performance. Scanning electron microscopy revealed distinctive structures in films with a nominal grain size of 9 μm, influencing grinding efficiency and surface quality. Mechanical binder removal and confocal microscopy provided insights into abrasive grain embedding. The study extended to the analysis of microchips generated during the lapping film finishing process, emphasizing their size, distribution, and impact on tool wear. The absence of spherical chips in the RG7 bronze alloy surface finishing process was noted. Furthermore, the study delved into machined surface roughness parameters, chip segmentation frequency, and machining trace width. Results indicated variations in surface characteristics based on lapping film grade, emphasizing the importance of optimal film selection for desired finishes in diverse applications.

- SEM images of lapping films with different nominal grain sizes (9 µm, 12 µm, and 30 µm) revealed variations in the machined zones and tool surfaces after processing. The distinct characteristics of the lapping film surfaces at various grain sizes emphasized the influence of film grades on resulting surface morphology. Microchips resulting from the surface finishing process were observed to be significantly smaller than the abrasive grains on the tool surface.
- Wear zones around abrasive aggregates were observed on 12LF and 9LF films, indicating a potential weakening of cutting capabilities and the risk of damaging the machined surface. In contrast, the absence of wear zones on the 30LF film suggested a more efficient evacuation of microchips, contributing to a controlled finishing process. Tool wear, manifested as clogging around abrasive aggregates, resulted in short, densely arranged chips near the highest peaks.
- Analysis of tool surfaces indicated that abrasive grains were exposed from beneath a thin binder layer just before the micromachining process, emphasizing the importance of the initial cutting conditions.
- Unusual agglomerates formed among finishing products due to high temperatures and chip rolling, resulting in fused structures. This phenomenon highlighted the influence of temperature on chip behavior during the finishing process.
- Examination of microchips revealed a segmented structure with diverse shapes and thicknesses. The presence of extremely thin chips with a segmented structure indicated a connection with high material separation speed. The analysis of chip segment thickness allowed for the calculation of chip segmentation frequency, reaching megahertz levels.
- Surface roughness parameters (Sp, Sv, Sz, Sa) were used to assess the quality of machined surfaces after lapping film processing. The results showed a decrease in parameters after each film application, with the final operation eliminating machining traces from previous treatments. The study explored the relationship between material removal rate, chip width, and lapping film characteristics.
- Despite extensive examinations, no occurrence of spherical chips or microspheres was observed during lapping film processing. The absence of spherical chips was attributed to the controlled smoothing of the RG7 bronze alloy, indicating specific temperature conditions, where the processing temperature did not exceed the melting temperature of the material.
- The study revealed diverse chip structures based on electrocorundum grain shapes, cutting edges, and chip thicknesses. The presence of jagged edges, segmented chips, and variations in chip width and shape highlighted the complexity of chip formation.

## Figures and Tables

**Table 1:** Machining conditions for the experiments.

Workpiece material	Tin Bronze Alloy (CuSn7Zn4Pb6/RG7)
Pressure roll hardness	50°Sh
Pressure force	50 N
Tool speed	160 mm/min
Workpiece speed	105 m/min
Oscillation frequency	80 Hz

**Table 2:** Descriptive statistics of research results on the thickness of the chip segment depending on the nominal size of the lapping film grain.

Chip Segment Thickness [nm]				
Lapping Film	Mean	Minimum	Maximum	Standard Deviation
LF9	156.54	119	199	22.69
LF12	152.68	111	198	17.30
LF30	197.65	140	286	36.81

**Table 3:** Chip segmentation frequency.

Chip Segmentation Frequency [MHz], the Frequency Determined within the Range of 5 to 15 Degrees of Tangential Grain Cutting Angle:	
Lapping Film	*f_s_*
LF9	0.975–2.893
LF12	0.999–2.966
LF30	0.772–2.291

**Table 4:** Descriptive statistics of research results on the chip width depending on the nominal size of the lapping film grain.

Chip Width [µm]				
Lapping Film	Mean	Minimum	Maximum	Standard Deviation
LF9	0.813	0.309	2.06	0.350
LF12	0.889	0.331	2.41	0.441
LF30	2.429	0.934	4.41	0.941

**Table 5:** Height parameters for describing surface roughness determined according to ISO 25178 standard.

Parameters for Assessing Surface Roughness [µm]				
Parameter [Unit]	Mean	Standard Deviation	Minimum	Maximum
LF9				
Sp [µm]	0.1071	0.03957	0.0660	0.1980
Sv [µm]	0.6714	0.1970	0.4311	0.9710
Sz [µm]	0.7785	0.2081	0.5117	1.084
Sa [µm]	0.01304	0.002492	0.01057	0.01967
LF12				
Sp [µm]	0.126	0.014	0.092	0.149
Sv [µm]	0.565	0.131	0.360	0.818
Sz [µm]	0.692	0.137	0.482	0.967
Sa [µm]	0.020	0.003	0.016	0.024
LF30				
Sp [µm]	0.1971	0.08096	0.1195	0.3965
Sv [µm]	0.7009	0.2127	0.3427	1.082
Sz [µm]	0.8980	0.2677	0.4626	1.406
Sa [µm]	0.02677	0.003374	0.02284	0.0337

**Table 6:** The width of the machining trace determined via the watershed segmentation method according to ISO 16610-45.

Parameter [Unit]	Mean	Standard Deviation	Minimum	Maximum
LF9				
AR [µm]	1.0001	0.1135	0.7425	1.17
LF12				
AR [µm]	1.0832	0.1773	0.7595	1.214
LF30				
AR [µm]	1.1898	0.0914	1.076	1.355

## Data Availability

Data are contained within the article.

## References

[1] Shan K., Zhang Y., Lan Y., Jiang K., Xiao G., Li B. (2023). Surface Roughness Prediction of Titanium Alloy during Abrasive Belt Grinding Based on an Improved Radial Basis Function (RBF) Neural Network. Materials.

[2] Serpin K., Mezghani S., El Mansori M. (2015). Multiscale assessment of structured coated abrasive grits in belt finishing process. Wear.

[3] Rech J., Kermouche G., Claudin C., Khellouki A., Grzesik W. (2008). Modelling of the residual stresses induced by belt finishing on a AISI52100 hardened steel. Int. J. Mater. Form..

[4] Wang W., Salvatore F., Rech J., Li J. (2018). Comprehensive investigation on mechanisms of dry belt grinding on AISI52100 hardened steel. Tribol. Int..

[5] Huang X., Guo Y., Guo W., Qi B., Ren X., Chai Z., Chen X. (2023). Comprehensive investigations into the force and thermal characteristics of belt grinding Inconel 718 under constant normal forces. J. Manuf. Process..

[6] Zhang Y., Xiao G., Zhou K., Zhu B., Gao H., Huang Y. (2023). Two-Phase fatigue life prediction method based on scSE U-net algorithm for abrasive belt grinding of titanium alloy. J. Mater. Process. Technol..

[7] Liu Y., Song S., Zhang Y., Li W., Xiao G. (2021). Prediction of surface roughness of abrasive belt grinding of superalloy material based on rlsom-rbf. Materials.

[8] Chen X., Dai Y., Hu H., Tie G., Guan C. (2019). Research on high precision and deterministic figuring for shaft parts based on abrasive belt polishing. Materials.

[9] Mezghani S., El Mansori M., Zahouani H. (2009). New criterion of grain size choice for optimal surface texture and tolerance in belt finishing production. Wear.

[10] Mezghani S., El Mansori M., Massaq A., Ghidossi P. (2008). Correlation between surface topography and tribological mechanisms of the belt-finishing process using multiscale finishing process signature. Comptes Rendus Mécanique.

[11] He Y., Xiao G., Li W., Huang Y. (2018). Residual stress of a TC17 titanium alloy after belt grinding and its impact on the fatigue life. Materials.

[12] Zhang B., Wu S., Wang D., Yang S., Jiang F., Li C. (2023). A review of surface quality control technology for robotic abrasive belt grinding of aero-engine blades. Meas. J. Int. Meas. Confed..

[13] Min K., Ni F., Liu H. (2023). Robotic abrasive belt grinding of complex curved blades based on a novel force control architecture integrating smooth trajectories. J. Manuf. Process..

[14] Khellouki A., Rech J., Zahouani H. (2010). The effect of lubrication conditions on belt finishing. Int. J. Mach. Tools Manuf..

[15] Mezghani S., El Mansori M. (2008). Abrasiveness properties assessment of coated abrasives for precision belt grinding. Surf. Coat. Technol..

[16] Serpin K., Mezghani S., El Mansori M. (2015). Wear study of structured coated belts in advanced abrasive belt finishing. Surf. Coat. Technol..

[17] Zou L., Liu X., Huang Y., Fei Y. (2019). A numerical approach to predict the machined surface topography of abrasive belt flexible grinding. Int. J. Adv. Manuf. Technol..

[18] Kacalak W., Tandecka K., Mathia T.G. (2016). A method and new parameters for assessing the active surface topography of diamond abrasive films. J. Mach. Eng..

[19] Sadeghifar M., Sedaghati R., Jomaa W., Songmene V. (2018). A comprehensive review of finite element modeling of orthogonal machining process: Chip formation and surface integrity predictions. Int. J. Adv. Manuf. Technol..

[20] Hamdi A., Merghache S.M., Fernini B., Aliouane T. (2021). Influence of polymer contacting rollers on surface texture finish in the belt grinding process. Int. J. Adv. Manuf. Technol..

[21] Perçin M., Aslantas K., Ucun I., Kaynak Y., Çicek A. (2016). Micro-drilling of Ti-6Al-4V alloy: The effects of cooling/lubricating. Precis. Eng..

[22] Yip W.S., To S. (2019). Reduction of Minimum Cutting Thickness of Titanium Alloys in Micro Cutting by a Magnetic Field Assistance. IEEE Access.

[23] Liu D., Ni C., Wang Y., Zhu L. (2024). Review of serrated chip characteristics and formation mechanism from conventional to additively manufactured titanium alloys. J. Alloys Compd..

[24] Barry J., Byrne G., Lennon D. (2001). Observations on chip formation and acoustic emission in machining Ti-6Al-4V alloy. Int. J. Mach. Tools Manuf..

[25] Sutter G., List G. (2013). Very high speed cutting of Ti-6Al-4V titanium alloy—Change in morphology and mechanism of chip formation. Int. J. Mach. Tools Manuf..

[26] Liu H., Zhang J., Xu X., Zhao W. (2018). Experimental study on fracture mechanism transformation in chip segmentation of Ti-6Al-4V alloys during high-speed machining. J. Mater. Process. Technol..

[27] Mathia T.G., Pawlus P., Wieczorowski M. (2011). Recent trends in surface metrology. Wear.

[28] Kubiak K.J., Wilson M.C.T., Mathia T.G., Carval P. (2011). Wettability versus roughness of engineering surfaces. Wear.

[29] (2021). Geometrical Product Specifications (GPS): Surface Texture: Areal—Part 2: Terms, Definitions and Surface Texture Parameters.

[30] (2015). Geometrical Product Specifications (GPS): Filtration—Part 45: Morphological Profile Filters: Segmentation.

[31] Szada-Borzyszkowska M., Kacalak W., Banaszek K., Borkowski P.J., Szada-Borzyszkowski W. (2023). Analysis of the pulsating properties of a high-pressure water jet generated in a self-excited head for erosion processing. Arch. Civ. Mech. Eng..

[32] Ye G. (2023). The formation mechanism of discontinuously segmented chip in high-speed cutting of Ti-6Al-4V. Int. J. Adv. Manuf. Technol..

[33] Kacalak W., Rypina Ł., Tandecka K. (2015). Evaluation of Micromachining Processes Using Data in the Format and Geometric Characteristics of Micro-Chips. J. Mach. Eng..

[34] Kouadri S., Necib K., Atlati S., Haddag B., Nouari M. (2013). Quantification of the chip segmentation in metal machining: Application to machining the aeronautical aluminium alloy AA2024-T351 with cemented carbide tools WC-Co. Int. J. Mach. Tools Manuf..

[35] Kacalak W., Rypina Ł., Królikowski T. (2015). Influence of analysis of features geometrical abrasive grains stress, strain and displacement of material in zone microgrinding. Mechanik.

[36] Devotta A., Beno T., Siriki R., Löf R., Eynian M. (2017). Finite Element Modeling and Validation of Chip Segmentation in Machining of AISI 1045 Steel. Procedia CIRP.

[37] Zanger F., Kacaras A., Bächle M., Schwabe M., León F.P., Schulze V. (2018). FEM simulation and acoustic emission based characterization of chip segmentation frequency in machining of Ti-6Al-4V. Procedia CIRP.

[38] Sahoo P., Banerjee N., Singh R.K. (2023). Modeling and analysis of chip segmentation in micro-cutting of Zr-based bulk metallic glass (BMG). Manuf. Lett..

[39] Nguyen V., Fernandez-Zelaia P., Melkote S.N. (2017). PVDF sensor based characterization of chip segmentation in cutting of Ti-6Al-4V alloy. CIRP Ann.-Manuf. Technol..

[40] Carvalho S., Horovistiz A., Davim J.P. (2023). Morphological characterization of chip segmentation in Ti-6Al-7Nb machining: A novel method based on digital image processing. Meas. J. Int. Meas. Confed..

[41] Siju A.S., Jose S., Waigaonkar S.D. (2022). Experimental analysis and characterisation of chip segmentation in dry machining of Ti-6Al-4V alloy using inserts with hybrid textures. CIRP J. Manuf. Sci. Technol..

[42] Joshi S., Pawar P., Tewari A., Joshi S.S. (2014). Effect of β phase fraction in titanium alloys on chip segmentation in their orthogonal machining. CIRP J. Manuf. Sci. Technol..

[43] Chen G., Caudill J., Ren C., Jawahir I.S. (2022). Numerical modeling of Ti-6Al-4V alloy orthogonal cutting considering microstructure dependent work hardening and energy density-based failure behaviors. J. Manuf. Process..

[44] Kacalak W., Lipiński D., Szafraniec F., Zawada-Tomkiewicz A., Tandecka K., Królczyk G. (2020). Metrological basis for assessing the state of the active surface of abrasive tools based on parameters characterizing their machining potential. Meas. J. Int. Meas. Confed..

[45] Long X., Chong K., Su Y., Chang C., Zhao L. (2023). Meso-scale low-cycle fatigue damage of polycrystalline nickel-based alloy by crystal plasticity finite element method. Int. J. Fatigue.

